# Energy reserves and respiration rate in the earthworm *Eisenia andrei* after exposure to zinc in nanoparticle or ionic form

**DOI:** 10.1007/s11356-019-05753-3

**Published:** 2019-06-26

**Authors:** Zuzanna M. Świątek, Agnieszka J. Bednarska

**Affiliations:** 1 Institute of Environmental Sciences, Jagiellonian University, Gronostajowa 7, 30-387 Kraków, Poland; 2 Institute of Nature Conservation, Polish Academy of Sciences, Mickiewicza 33, 31-120 Kraków, Poland

**Keywords:** Energy budget, Zinc, Nanoparticle, Soil, Earthworm, Metabolism

## Abstract

The energy budget is an indicator of an organism’s overall condition. Changes in energy reserves and/or energy consumption have been used as biomarkers of toxic stress. To understand the effects of different forms and concentrations of Zn and the costs of effective Zn regulation by the earthworm *Eisenia andrei*, we performed a toxicokinetic experiment in which individuals were sampled over time to determine the available energy reserves (total carbohydrate, protein, and lipid content), energy consumption (measured at the cellular level and as the whole-animal respiration rate), and internal Zn concentration. The earthworms were exposed to ZnCl_2_ or zinc nanoparticles (ZnO-NPs) in Lufa 2.2 soil for 21 days (contamination phase), followed by 14 days of elimination in clean soil (decontamination phase). Carbohydrates were the only energy reserves with significantly lower levels following ZnO-NP 1000 treatment than following other treatments (*p* ≤ 0.00001) in the contamination phase. The total available energy reserves and protein content did not differ among treatments, but a significant effect of exposure time was observed (*p* ≤ 0.0001). Exposure to Zn (both ions and NPs) increased energy consumption at the cellular level, reflecting the high energy demand of the stress response. The results indicated that *E. andrei* can regulate internal Zn concentrations efficiently, regardless of form or concentration, without considerable impact on energy reserves or respiration rate.

**Electronic supplementary material:**

The online version of this article (10.1007/s11356-019-05753-3) contains supplementary material, which is available to authorized users.

## Introduction

Nanoparticles (NPs) exhibit eminent technological potential that stems from the specific physicochemical properties of these particles. NPs are used in various industries, such as the chemical, automotive, cosmetic, textile, and electronic industries (Hansen et al. 2016). Zinc oxide NPs (ZnO-NPs) are among the most widely used NPs and have been successfully incorporated into semiconductors, electronic sensors, solar voltaic cells, and electrical and optical devices. Moreover, because of the UV absorption and antimicrobial and antifungal properties exhibited by these particles, ZnO-NPs are commonly utilized in pharmaceuticals and personal care products (Stark et al. 2015). NPs also have great potential applications in agriculture and the food industry via deliberate application in the form of plant protection products, fertilizers (Khot et al. 2012), or supplements (Swain et al. 2016). The widespread production, usage, and application of these particles make NPs a group of pollutants that have a potential impact on human health and the environment, but this impact has not been fully elucidated (Lead et al. 2018). According to the latest report by the United Nations Environment Programme, nanomaterials are one of the six key emerging issues affecting the planet (UNEP 2017). Although the quantities of NP that reach the soil are estimated to be very low (Sun et al. 2014), the production and usage of NPs are constantly increasing (Part et al. 2018). Therefore, it is important to assess whether the entry of NPs into the soil can affect soil organisms and pose a risk to soil communities.

It has been already recognized that upon reaching the soil, ZnO-NPs may have a negative impact on soil microorganisms, invertebrates, and plants (Hou et al. 2018). Most studies have found, however, that ZnO-NPs are less toxic than zinc ions to soil-dwelling organisms (Garcia-Gomez et al. 2015), including earthworms (Kwak and An 2015). Initially, the dissolution of zinc ions rather than the direct impact of NPs was proposed as the mechanism underlying the toxicity of ZnO-NPs (Kool et al. 2011). However, Hua et al. (2014) demonstrated that the toxicity of ZnO-NPs against zebrafish (*Danio rerio*) embryos resulted from the combined effect of the particles themselves and the dissolved ions released from the NPs. Recently, published studies on terrestrial organisms have revealed that the toxicity of ZnO-NPs against plants, microbial communities (Judy et al. 2015), and earthworms (Lahive et al. 2017) may have been underestimated. For example, Lahive et al. (2017) showed that *Eisenia fetida* reproduction was affected to a greater degree after exposure to engineered NPs (ZnO-NPs, TiO_2_-NPs, Ag-NPs) than after exposure to metal salts when earthworms were exposed to soils amended with sewage sludge from a wastewater treatment plant treated with NPs or metal salts.

Responding to stress caused by exposure to a toxicant is usually associated with energetically costly processes of detoxification, e.g., synthesis of metallothionein-like proteins or storage of metals in granules. The energy budget of an organism is fixed, and therefore, the energetic expense of detoxification diminishes the amount of energy available for other maintenance costs, such as those of growth and reproduction (Calow 1991). This reduction in resources can be determined either indirectly, e.g., by measuring reproduction (Organisation for Economic Co-Operation and Development 2004), or by direct determination of available energy reserves (De Coen and Janssen 1997). Reproduction is recognized as an ecologically relevant and sensitive toxicity endpoint and has already been used to compare the toxicity of ZnO-NPs with than of Zn ions in earthworms, but with ambiguous results (Lahive et al. 2017; Romero-Freire et al. 2017). No studies have been performed to determine the changes in the energy budget of earthworms exposed to ZnO-NPs. In the energy budget approach, available energy reserves (Ea), measured as a sum of the carbohydrate, lipid and protein content, and energy consumption (Ec), assessed by measuring the electron transport system (ETS) activity, are determined at the cellular level. This approach has been applied by several researchers to measure the effects of metal stress on soil invertebrates (Bednarska et al. 2013), but only a few studies have examined the effects of NPs (e.g., TiO2-nano, CuO-nano, Ag-nano) (Khalil 2015; Gomes et al. 2015a). Moreover, in most studies that have focused on energy budget and NP toxicity, the internal metal concentration was not measured, although it seems that depletion of energy reserves could be associated with excretion of metals (Holmstrup et al. 2011).

Oxygen consumption, measured as the whole-body respiration rate, is another indicator of metabolic changes that has been successfully used in studies on the toxicity of metals (Khan et al. 2007) and NPs (Liang et al. 2017) in earthworms. The general prediction of models involving metabolically costly physiological responses is that the metabolic rate should increase with increasing intoxication (exposure time and/or concentration) until irreversible pathological effects impair metabolism itself (Calow 1989). To provide an in-depth view of the sublethal effects of toxic stress, differences in actual oxygen consumption (whole-body respiration rate as the equivalent of metabolic rate) can be related to similar differences in potential oxygen consumption, a biochemical measure of potential metabolic activity, using ETS (Martinez 1992).

Our previous study on the toxicokinetics of ZnO-NPs and zinc ions in the earthworm *Eisenia andrei* revealed that Zn was efficiently regulated by the earthworms, regardless of the form of this metal present in the soil, and the differences in Zn assimilation and elimination rates between treatments were connected with exposure concentrations rather than the form of Zn (Świątek et al. 2017). To understand the effects of different forms and concentrations of Zn on the energy budget of *E. andrei* and to determine the energy costs of the detoxification process, we designed a toxicokinetic experiment in which earthworms were sampled periodically to measure energy reserves (total lipid, carbohydrate, and protein content), energy consumption (measured both at the cellular level and as the whole-body respiration rate), and internal Zn concentrations after exposure to Zn present in soil in the form of NPs (ZnO-NPs) or ions (ZnCl_2_).

## Materials and methods

### Test species

Earthworms of the species *E. andrei* were obtained from a laboratory culture. The earthworms were fed with horse manure free of any pharmaceuticals and cultured at 20 °C in darkness. The experiment used 4-month-old adult individuals with well-developed clitella.

### Chemicals

Uncoated ZnO nanopowder with an advertised 25-nm particle size was purchased from PlasmaChem GmbH, and ZnCl_2_ was purchased from Merck Group. Other chemicals used in the present study were of analytical grade unless otherwise stated. Trichloroacetic acid (TCA), Bradford reagent, bovine serum albumin (BSA), glucose standard solution, iodonitrotetrazolium chloride (INT), β-nicotinamide adenine dinucleotide (NADH), β-nicotinamide adenine dinucleotide phosphate (NADPH), polyvinylpyrrolidone (PVP), tris(hydroxymethyl)aminomethane (Tris base), t-octylphenoxypolyethoxyethanol (Triton™ X-100), and glyceryl tripalmitate were purchased from Sigma-Aldrich®. CHCl_3_ (chloroform), CH_3_OH (methanol), C_6_H_6_O (phenol), NaOH, HCl, H_2_SO_4_, MgSO_4_, and HNO_3_ (69.9–70.0%; Baker Instra-Analyzed) were purchased from Avantor Performance Materials S.A., and H_2_O_2_ (30.0%; Baker Analyzed) was purchased from WITKO®.

### Nanoparticle characterization

The size measurements and morphological analyses of the NPs were performed by transmission electron microscopy (TEM) using a high-resolution analytical transmission electron microscope (FEI Tecnai Osiris) equipped with an X-FEG Schottky field emitter (200 kV). The Z-contrast images were acquired using a high-angle annular dark-field detector in scanning TEM mode. Prior to microscopic analysis, the samples were dispersed in ethanol and dropped on a lacey carbon film supported on a copper grid (Ted Pella Inc., 300 mesh). The average primary particle diameters (± SD) were determined by measurement of the particles (*N* = 500) in random fields of ten images using the ImageJ software package. The hydrodynamic diameters and zeta potentials of the particles were determined by dynamic light scattering using a Malvern Zetasizer Nano ZS (Malvern, UK) in deionized water (nominal concentration 5 mg Zn ml^−1^). The size distribution was measured by intensity.

### Soil spiking procedure

Standardized Lufa 2.2 loamy sand soil (Lufa-Speyer 2.2, Germany, 2016) was used, with a reported pH_CaCl2_ of 5.4 ± 0.2 (mean ± SD), total organic carbon content of 1.61 ± 0.15%, cation exchange capacity of 9.7 ± 0.4 meq 100 g^−1^, and maximum water holding capacity (WHC) of 44.8 ± 2.9% (*w*/*w*). Two concentrations of ZnO-NPs (nominal: 500 and 1000 mg Zn kg^−1^ dry soil, designated as ZnO-NP 500 and ZnO-NP 1000, respectively), two concentrations of ZnCl_2_ (nominal: 250 and 500 mg Zn kg^−1^ dry soil, designated as ZnCl_2_ 250 and ZnCl_2_ 500, respectively), and one control with ca. 27 mg Zn kg^−1^ dry soil, of the natural Zn levels in soil, were studied. The chosen concentrations corresponded to the EC_25_ and EC_50_ for earthworm reproduction (Heggelund et al. 2014) and represented low and medium values of typical Zn contamination in urban areas (Stafilov et al. 2010; Gonzalez-Alcaraz et al. 2018). For equilibration, the soil was spiked 7 days before exposure. For convenience, the test compounds were introduced into the soil using different methods, but the mode of application does not influence the distribution of NPs (Waalewijn-Kool et al. 2012). Zinc chloride (ZnCl_2_) was applied as an aqueous solution, and NPs (ZnO) were dosed as dry powder, followed by the addition of sufficient water to attain a soil moisture content that was equivalent to 50% of the WHC. The spiking procedure is fully described in Świątek et al. (2017). After spiking, to achieve homogenous distribution, each batch of soil was homogenized carefully.

### Experimental design

The test was performed by following the modified OECD Guideline 317 (Organisation for Economic Co-Operation and Development 2010), with 21 days of exposure in Zn-contaminated soil (contamination phase, also described in the literature as the uptake phase), followed by 14 days of elimination in control soil (decontamination phase, also described in the literature as the elimination phase). Five replicates (round plastic containers filled with approximately 60 g of wet soil) were prepared per sampling point for each Zn treatment and control (i.e., animals kept in uncontaminated soil throughout the experiment). Food was added at the beginning of each phase by mixing 7 mg dry weight of horse dung per 1 g dry weight of soil prior to introducing the soil into the test containers. Food was added to ensure earthworm growth and well-being, as we expected that in an experiment lasting 35 days, food deprivation might act as an additional stressor (Spurgeon et al. 2003). Before starting the experiment, earthworms were placed in empty Petri dishes for 24 h to void the gut contents of the worms, washed in tap water, and then weighed to the nearest 0.0001 g and randomly assigned to treatments, one individual per container. Containers were kept at 20 °C and 75% relative humidity (RH) under a 16:8 (L:D) h cycle. Once a week, the soil moisture content was checked by weighing the containers, and the moisture lost was replenished with tap water when necessary. The containers were also aerated by this procedure. Before starting the exposure (day 0) and after 1, 2, 4, 7, 14, 17, and 21 days (contamination phase) and 22, 23, 28, and 35 days (decontamination phase), five individuals were sampled from each Zn treatment and control. At each sampling point, the collected earthworms were rinsed with tap water and blotted dry on filter paper. Then, the animals were kept individually for 9 h in Petri dishes lined with moistened filter paper to void the gut content of the worms. Afterwards, the worms were placed in respiratory chambers for an additional 15 h for measurement of respiration. Twenty four hours after sampling from the soil, (i.e., after 24 h of depuration), the animals were rinsed, blotted dry, weighed (to the nearest 0.0001 g), frozen in liquid nitrogen, and stored at − 80 °C until further analysis.

### Whole-body respiration rate

To ensure that they did not escape during measurement, the earthworms were placed individually in 6-mL tubes closed with breathable mesh, and then, each tube was placed in a 50-mL glass flask connected to a 30-channel computer-controlled closed-circuit Micro-Oxymax respirometer (Columbus Instruments, USA). To reduce desiccation, a small piece of filter paper moistened with tap water was placed in each tube. The whole-body respiration rate was measured for 15 h at 3-h intervals at 20 °C and 16:8 (L:D) h. The respiration rate was measured as oxygen consumption per hour per earthworm and then recalculated per gram body mass for data analysis (μL O_2_ g^−1^ h^−1^). Prior to data analysis, the first measurement point (the first 3-h interval) for each individual was excluded from the data because the change in the environment and handling stress might have temporarily provoked abnormal activity and respiration rates. Measurements were not corrected for oxygen consumption by microbes present in the flask and/or earthworm feces, as previous studies showed that the contribution of microbial respiration is negligible (Uvarov and Scheu 2004; Tang et al. 2016).

### Energy reserves and energy consumption analysis

Available energy reserves (Ea) were measured by quantifying the total lipid, protein, and carbohydrate content, and cellular energy consumption (Ec) was assessed by measuring ETS activity as described by De Coen and Janssen (1997), Bednarska et al. (2013), and Bednarska and Stachowicz (2013) with some modifications for earthworms. Earthworms were homogenized on ice using a mechanical Omni tissue homogenizer (TH220-PCR), and measurements were performed on 96-well plates (Sarstedt) using a μQuant spectrophotometer (Bio-Tek Instruments).

Each sample was analyzed in triplicate or quadruplicate. Earthworms were homogenized on ice in homogenization buffer (0.08 M Tris base-HCl (pH 8.5), 15% (*w*/*v*) PVP, 153 μM MgSO_4_, and 0.2% (*w*/*v*) Triton X-100) in a total volume of 600 μL. The homogenate of each earthworm was divided into two parts: 150 μL (i.e., ¼ earthworm) was used for energy consumption (Ec) measurement, and 450 μL (i.e., ¾ earthworm) was used for both available energy (Ea) and Zn concentration analysis. For Ea determination, the homogenate was diluted with demineralized water and used for measurement of total lipid, protein, and carbohydrate levels. For total lipid content measurements, 500 μL of chloroform, 500 μL of methanol, and 250 μL of demineralized water were added to 200 μL of diluted homogenate. After centrifugation (1000×*g*, 5 min, 20 °C), the top phase was removed, and 500 μL of H_2_SO_4_ was added to the remaining lipid extract. After mixing, the sample was incubated for 15 min at 200 °C. Then, the sample was cooled to room temperature and diluted with 1.5 mL of demineralized water. The total lipid content was determined by measuring the absorbance at 400 nm using glyceryl tripalmitate as a standard. For the total protein and carbohydrate content, 100 μL of 15% TCA was added to 300 μL of diluted homogenate, and the sample was incubated at − 20 °C for 10 min. Then, the sample was centrifuged (1000×*g*, 4 °C, 10 min), and the supernatant was collected for carbohydrate measurement, whereas the pellet was resuspended in 500 μL of NaOH, incubated at 60 °C for 30 min, and neutralized with 300 μL of 1.67 M HCl. To determine the total protein content, the sample was incubated (25 °C, 15 min) with Bradford’s reagent, and the absorbance was measured at 600 nm using BSA as a standard. To determine the total carbohydrate content of the supernatant fraction, 5% phenol and H_2_SO_4_ were added to the supernatant at a 1:4:1 ratio. The sample was incubated for 30 min at room temperature and shaken during this period, and then, the absorbance was measured at 490 nm using a standard curve for glucose. The different energy reserves for each individual were converted into energetic equivalents using the energy of combustion (17.5 kJ/g glycogen, 24 kJ/g protein, 39.5 kJ/g lipids) and summed.

Energy consumption (Ec) was determined by measuring the ETS activity. The homogenate (150 μL, i.e., ¼ earthworm) was centrifuged (1000×*g*, 10 min, 4 °C) and diluted 5 times using ice-cold homogenization buffer. ETS was quantified by adding 150 μL of buffered substrate solution (0.13 mM Tris base-HCl (pH 8.5), 0.3% (*w*/*v*) Triton X-100, 1.7 mM NADH, 250 μM NADPH) to 50 μL of the resulting supernatant. To start the colorimetric reaction, 100 μL of reagent solution (INT) was added, and the absorbance was measured kinetically at 490 nm every 36 s for 3 min at 20 °C. Formazan production was determined by measuring the absorbance of the sample against the blank using *ɛ* = 15,900 M^−1^ cm^−1^. The oxygen consumption rate was determined from ETS based on the theoretical stoichiometric relationship, i.e., for each 2 μM formazan formed, 1 μM oxygen is consumed by the ETS. The quantity of oxygen consumed was expressed per gram body mass (μL O_2_ g^−1^ h^−1^).

### Physicochemical analysis

To analyze the Zn concentration, the earthworm tissue homogenates and soil samples (three per treatment) were dried at 105 °C for 24 h and weighed to the nearest 0.0001 g. Samples of homogenized earthworms were digested in 0.5 mL of boiling HNO_3_ (69.0–70.0%) and then diluted to 5 mL with 0.2% HNO_3_. Soil samples were digested in 10 mL of a 4:1 mixture of HNO_3_ and H_2_O_2_ and then supplemented with 30 mL of demineralized water. Zn concentrations in the solutions were measured using flame atomic absorption spectrometry (AAS) (Perkin Elmer AAnalyst 200) and expressed in mg kg^−1^ dry weight (dw). To determine analytical precision, three blanks and three samples of a certified reference material (for earthworms: Dolt-5 dogfish liver, National Research Council of Canada, with a certified Zn concentration of 105.3 ± 5.4 mg kg^−1^ (mean ± SD); for soil: loamy sand 10 (Sigma-Aldrich), with a certified Zn concentration of 203 ± 5.29 mg kg^−1^) were examined with the samples. The measured Zn concentrations in the reference materials were within ± 10% and ± 8.2% of the certified concentrations for the Dolt-5 dogfish liver and loamy sand 10, respectively.

Soil samples were additionally characterized for Zn concentration in water extracts and extracts after ultrafiltration at days 0, 7, 14, and 21. The water-extractable Zn concentration was measured with demineralized water (1:4 *w*/*v*). Samples were shaken for 2 h at 2000 rpm and filtered through cellulose acetate paper (Eurochem BGD). Zn concentration in ultrafiltrates, i.e., particle-free water extracts, were determined from an aliquot of the water extracts. After filtration through 0.45-μm syringe filters (Chemland), samples were centrifuged in a 3-kDa ultrafiltration device (Amicon Ultra-15 Filters, Millipore) for 45 min at 4000×*g*. The 3-kDa pore size is estimated to be 2.1 ± 1.3 nm (Van Koetsem et al. 2017); hence, it prevents the penetration of nanoparticles through the membrane and at the same time allows the penetration of ions. Then, the water extracts and ultrafiltrates were acidified with a drop of HNO_3_ and analyzed for Zn concentration using flame AAS (Perkin Elmer AAnalyst 200). To determine analytical precision, blanks, and standard solutions with known Zn concentrations were analyzed. The soil pH was measured potentiometrically with 0.01 M CaCl_2_ (1:5 *w*/*v*) at days 0, 7, 14, and 21. Soil samples were shaken for 2 h at 2000 rpm, and after allowing the floating particles to settle overnight, the pH was measured using a pH meter. The soil organic matter content was determined at days 0 and 21 as loss on ignition.

### Data handling and statistical analysis

The distributions of all the studied parameters were checked for normality with Shapiro–Wilk’s W test, and the homogeneity of variances was checked with Leaven’s test. If the criteria were not met, the data were either (log or square root) transformed, or a nonparametric test was used. The effect of exposure time on Zn concentration in water extracts and ultrafiltrates and the pH was tested using the Kruskal–Wallis test, and if significant differences were observed, a Bonferroni procedure was used to identify the pattern of differences among exposure days significant at the 95.0% confidence level. To verify that individuals assigned to different treatments did not differ in the initial (day 0) body mass, one-way ANOVA was performed. To check whether earthworms lost body mass during the experiment, the body mass change (BMC) index was calculated for each individual based on the mass of the depurated earthworms according to the following equation: BMC = (*M*_*n*_ – *M*_0_)/*M*_0,_ where *M*_*n*_ is the mass of an earthworm at sampling day *n* (g), and *M*_0_ is the initial mass (at day 0) of the same earthworm (g). The effect of treatment and time on the BMC index was tested with the Kruskal–Wallis test for each phase separately, and when significant differences were found, a Bonferroni procedure was used to identify the pattern of differences among treatments or sampling days significant at the 95% confidence level. The effect of treatment and sampling day on carbohydrates, proteins, lipids, Ea, Ec, and R was tested for each phase (contamination and decontamination) separately using two-way ANOVA, with body Zn concentration as a covariate. Day 0, which was common for all treatments, was excluded from ANOVA to allow for testing of the interaction between factors. Nonsignificant (p ≤ 0.05) interactions and/or covariates were removed from the model. Where significant differences were observed, a post hoc least squares difference (LSD) test was used to identify the pattern of differences among treatments and/or exposure days. To verify that each of the measured parameters changed after exposure and returned to the pre-exposure state, one-way ANOVA was performed for each treatment separately with time (limited to days 0, 21, and 35) as a factor. The data were analyzed statistically using Statgraphic Centurion XVI (StatPoint Technologies, Inc., version 18).

## Results

### ZnO nanoparticle characterization

TEM analysis indicated that the particles were spherical (Fig. S1), and the average primary particle diameter (± SD) was 23.5 ± 7.3 nm (the manufacturer-provided particle size was ca. 25 nm). High-angle annular dark-field imaging (Fig. S2) with energy-dispersive X-ray analysis (Fig. S3) confirmed the chemical composition of the ZnO-NPs. Dynamic light scattering measurements showed that the particle size changed as soon as the NPs were submerged in water and aggregates (ca. 860 nm) were formed. The size distribution and zeta potentials observed using dynamic light scattering measurements are presented in Table S1 and Fig. S4.

### Soil physicochemical properties

The measured Zn concentrations (mean ± SD) in the test soil were in accordance with the nominal concentrations (Table S2). The zinc concentration in the control soil was 27.2 ± 1.6 mg kg^−1^ dw. Different patterns of Zn partitioning were observed for ionic and NP treatments. The average Zn concentrations in the water extracts were lowest for the ZnCl_2_ 250 treatment (0.63 ± 0.1 mg L^−1^) and highest for the ZnO-NP 1000 treatment (2.11 ± 0.2 mg L^−1^). The zinc concentrations remained approximately constant throughout the contamination phase in the ZnCl_2_ 250 treatment, decreased in ZnCl_2_ 500 treatment and increased in both ZnO-NP treatments (Table S2). In the ZnCl_2_ 250 and ZnCl_2_ 500 treatments, the average Zn concentrations in the ultrafiltrates corresponded to 56–88% and 84–95% of the Zn in the water extracts, respectively. In the ZnO-NP 500 and ZnO-NP 1000 treatments, the Zn concentrations in the ultrafiltrates corresponded to 30–68% and 27–54% of the Zn in the water extracts (Table S3). There were significant differences in soil pH_CaCl2_ values between exposure days (Table S4). In general, the soil pH was lower in soils spiked with ZnCl_2_ and in the control than in soils spiked with ZnO-NPs. For the ZnO-NP treatments and the control, the pH first increased and then decreased, resulting in a significantly higher pH at day 7 than at day 21 (Table S4). In the ZnCl_2_ 500 treatment, the pH was similar at days 0 and 7 but then decreased and increased again, with significant differences between days 14 and 21. In the ZnCl_2_ 250 treatment, the pH was constant during the course of exposure (Table S4). The average soil organic matter content was similar for all treatments on day 0 (4.5 ± 0.1%) and day 21 (4.4 ± 0.1%).

### Earthworms

All the earthworms survived until the end of the experiment. The mean initial body mass (± SD) of the earthworms was 0.256 ± 0.04 g (*N* = 260), with no significant difference between treatments (*p* = 0.71). The effect of sampling day on the BMC index was significant in both the contamination (*p* ≤ 0.00001) and decontamination (*p* = 0.003) phases, showing that body mass increased with time (Fig. S5). The effect of treatment on the BMC index was significant in the contamination phase (*p* ≤ 0.00001) with earthworms exposed to ZnO-NP 1000 exhibiting lower BMC index values than those from other treatments. Although earthworms from the ZnO-NP 1000 treatment exhibited a BMC reduction in the contamination phase, these worms were able to gain weight in the decontamination phase (Fig. S5). In general, earthworms from all the treatments gained weight in the decontamination phase, but this weight gain was uneven among the different treatments (*p* ≤ 0.00001, Fig. S5).

### Energy reserves

**Fig. 1:**
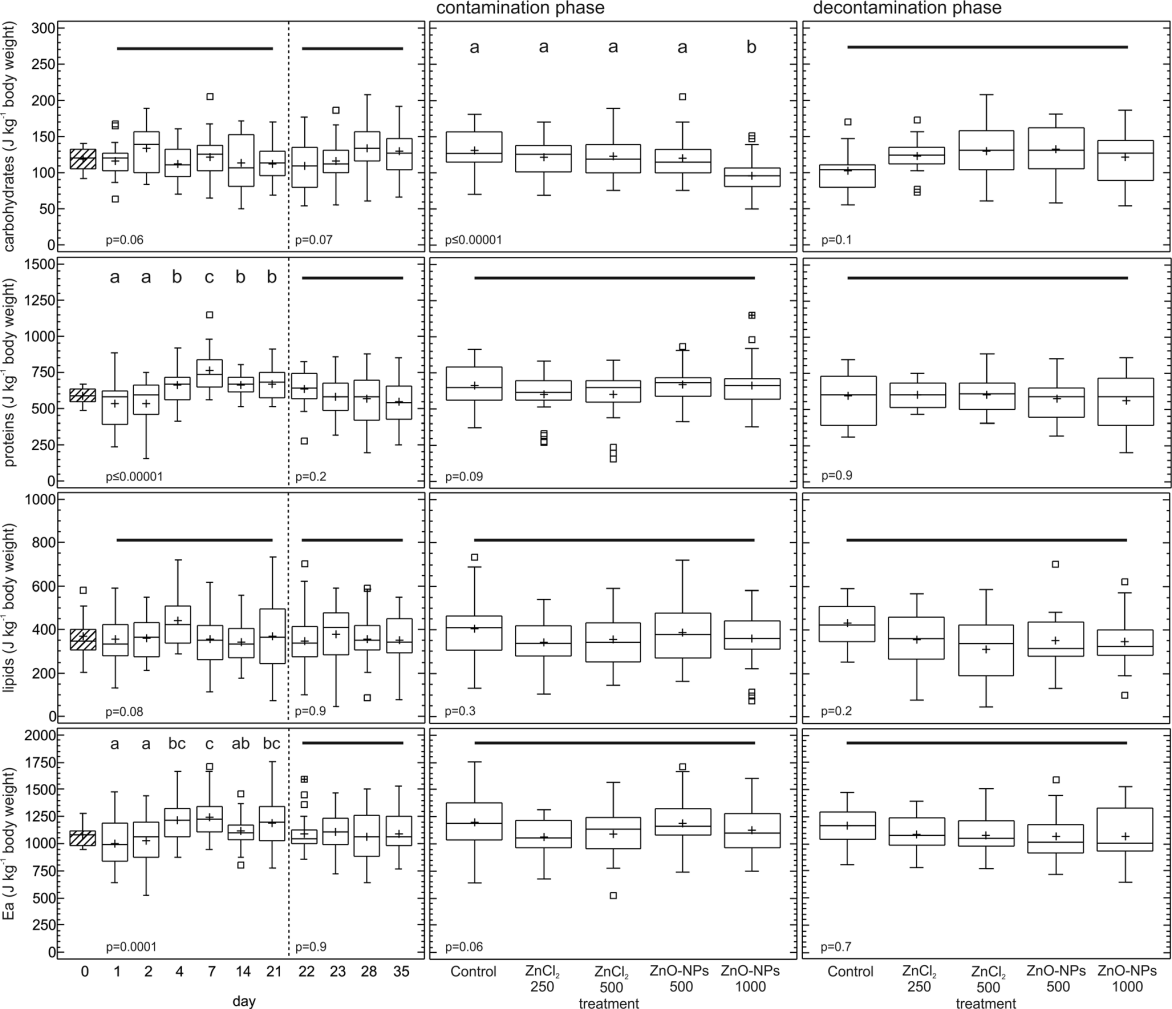
Effects of Zn on the available energy reserves (Ea), proteins, carbohydrates, and lipids in *Eisenia andrei* earthworms exposed to Lufa 2.2 soil contaminated with different effective concentrations (EC_25_ and EC_50_ for reproduction) of ZnO nanoparticles (NPs) or ions (ZnCl_2_). Boxes—lower and upper quartiles, whiskers—extend to the minimum and maximum values, plus sign—mean value, center line—median, empty squares (outliers)—between > 1.5 and 3 times the interquartile range, squares with crosses (far outliers)—more than 3 times the interquartile range. Shading indicates day 0, which was shared by all the treatments. The vertical broken line indicates the start of the decontamination phase. Different letters indicate significant differences (LSD, *p* < 0.05), and the solid horizontal line indicates that there was no difference between the treatments

The average total available energy reserves (Ea) of the earthworms (*N* = 10) at day 0 (before the exposure) consisted of 55% proteins (588.4 ± 55.1 J kg^−1^), 11% carbohydrates (119.3 ± 15,6 J kg^−1^), and 34% lipids (369.6 ± 109.2 J kg^−1^). In the contamination phase, the carbohydrate content was the only energy reserve component for which a highly significant effect of treatment was observed (*p* ≤ 0.00001): the earthworms from the ZnO-NP 1000 treatment had significantly lower levels of carbohydrates than those from other treatments (Fig. 1). Additionally, a marginally significant effect of exposure day on carbohydrate content was noted (*p* = 0.06). The protein contents did not differ significantly among treatments (*p* = 0.09), but a significant effect of sampling day was observed (*p* ≤ 0.00001), whereas neither treatment (*p* = 0.3) nor sampling day (*p* = 0.08) affected the lipid content (Fig. 1). The effect of treatment on Ea was almost significant (*p* = 0.06), and a significant effect of sampling day was observed (*p* = 0.0001). Neither treatment nor sampling day significantly affected Ea or any of the components in the decontamination phase (Fig. 1). There were no differences among days 0, 21, and 35 for either Ea or any of the energy reserve components (*p* > 0.06), except in the ZnO-NP 500 treatment, in which Ea was significantly higher at day 21 than at days 0 and 35 (*p* = 0.02).

### Energy consumption and whole-body respiration rate

**Fig. 2:**
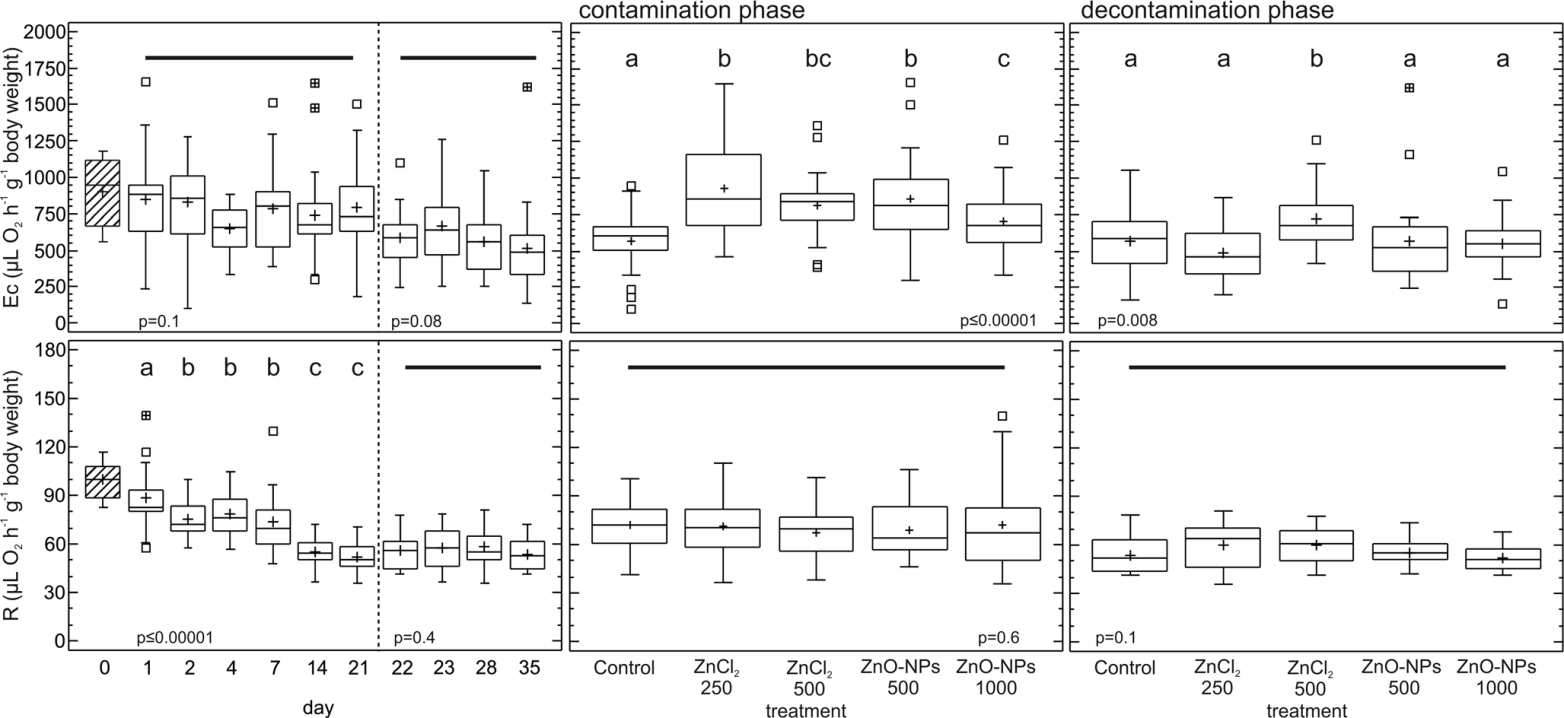
Effects of Zn on the energy consumption (Ec) and respiration rate (R) in *Eisenia andrei* earthworms exposed to Lufa 2.2 soil contaminated with different effective concentrations (EC_25_ and EC_50_) of ZnO nanoparticles (NPs) or ions (ZnCl_2_). Boxes—lower and upper quartiles, whiskers—extend to the minimum and maximum values, plus sign—mean value, center line—median, empty squares (outliers)—between > 1.5 and 3 times the interquartile range, squares with crosses (far outliers)—more than 3 times the interquartile range. Shading indicates day 0, which was shared by all the treatments. The vertical broken line indicates the start of the decontamination phase. Different letters indicate significant differences (LSD, *p* < 0.05). The solid horizontal line indicates that there was no difference between the treatments

Ec differed significantly among treatments in both the contamination (*p* ≤ 0.00001) and decontamination (*p* = 0.008) phases, with no effect of sampling day observed (*p* = 0.1 for the contamination phase and *p* = 0.08 for the decontamination phase) (Fig. 2), but there was a significant correlation with Zn concentration (as a covariate) in the decontamination phase (*p* = 0.02). In the contamination phase, the earthworms from all the Zn treatments had significantly higher Ec levels than those from the control, and at the same time, the Ec levels were significantly lower in the ZnO-NP 1000 treatment than in both the EC_25_ treatments (i.e., ZnO-NP 500 and ZnCl_2_ 250). In the decontamination phase, only earthworms exposed to the ZnCl_2_ 500 treatment had significantly higher Ec levels than those from other treatments. With regard to differences among days 0, 21, and 35, Ec was significantly lower at days 21 and 35 than at day 0 (*p* = 0.003) for the control, at day 35 than at days 0 and 21 (*p* = 0.0003) for ZnCl_2_ 250, and at day 35 than at day 0 (*p* = 0.004) for ZnO-NP 1000.

The whole-body respiration rate was similar for all the treatments in both phases of the experiment, and a gradual decrease in the respiration rate over time (*p* < 0.00001) was observed in the contamination phase (Fig. 2). For each studied treatment, the respiration rate was significantly lower on days 21 and 35 than on day 0 (*p* ≤ 0.00001), but no difference was observed between days 21 and 35.

## Discussion

### Soil properties

A variety of extraction methods can be used to extract trace elements from soil (Peijnenburg et al. 2007), but there are no specific methods recommended for the extraction of NPs, and different methods have been proposed in the literature (Rodrigues et al. 2016). In this study, we used a water extraction method to assess the availability of Zn during the contamination phase. It is generally agreed that this method enables the extraction of the most labile pool of Zn, which is easily accessible to soil-dwelling organisms (Garcia-Gomez et al. 2014). It has been suggested that the energy reserves of earthworms respond to easily extractable metals to a greater extent than to strongly bound metals (Beaumelle et al. 2014). The most labile pool of Zn can be absorbed via the epidermis (dermal uptake), which was estimated to be ca. 5–70% of the total Zn uptake in earthworms depending on the Zn concentration in soil (Vijver et al. 2003; Laycock et al. 2016), or via the gastrointestinal tract (oral uptake) with whole soil matrix. Although a relatively small percentage of the total Zn content was extracted for both the ionic and NP treatments, differences in Zn partitioning in the soil were observed for the studied forms of Zn. Changes in Zn partitioning over time were observed for both the EC_50_ treatments (ZnCl_2_ 500 and ZnO-NP 1000) and the ZnO-NP 500 treatment, but the changes proceeded in different directions: the Zn concentration in the extract decreased with time in the ionic treatment and increased in both NP treatments (Tables S2 and S3). This result is consistent with the study by Romero-Freire et al. (2017), in which an increase in Zn concentration in porewater was observed for NP treatments, while a decrease was observed for ZnCl_2_ treatment during aging (1, 3, 56, and 168 days). Several environmental processes may affect the partitioning of Zn in NP or ionic forms in soils (Cornelis et al. 2014). The decrease in Zn concentration in the ionic treatment may be associated with the predominance of metal sorption to soil granules and mineral precipitation. In NP treatments, dissolution is most likely responsible for the increase in Zn concentration in water extracts (Cornelis et al. 2014). Zn partitioning is inextricably linked to soil acidity (Sauvé et al. 2000), which, in turn, depends on the form of Zn added to the soil. In this study, a relatively low pH was observed for ionic treatments, and a relatively high pH was observed for NP treatments, which is consistent with the results of other studies (Kool et al. 2011; Garcia-Gomez et al. 2015).

### Earthworms

In the present study, Zn toxicity in the form of NPs and ions was investigated via a toxicokinetic experiment at two effective concentrations (EC_25_ and EC_50_ for reproduction). This framework enabled us to track changes between different treatments and sampling days during the contamination or decontamination phases. The energy reserves of *E. andrei* measured before the experiment (day = 0) consisted mainly of proteins (55%) and lipids (34%), with carbohydrates representing the smallest fraction (11%). These findings are consistent with those of other studies on different earthworm species (Laverack 1963; Beaumelle et al. 2014). Although the contribution of carbohydrates to the total energy reserves was the lowest, this component of Ea was the only one for which a clear effect of treatment was observed in the contamination phase. Carbohydrates are the first energy source mobilized by organisms under toxic stress (Moolman et al. 2007), and rapid mobilization of carbohydrates has been previously demonstrated in earthworms exposed to metals (Bundy et al. 2008; Holmstrup et al. 2011; Khalil 2015). For example, after seventy days of exposure to sublethal Cu concentrations (10–480 mg kg^−1^), increased transcript levels of carbohydrate-metabolizing enzymes (maltase-glucoamylase, mannosidase) followed by increased use of glucose and mannose have been observed in the earthworm *Lumbricus rubellus* (Bundy et al. 2008). A negative correlation between glycogen content and the concentrations of Ni, Al, and Zn was also observed in *Dendrobaena octaedra* earthworms after 28 days of exposure to soils from differently contaminated areas (Holmstrup et al. 2011). Khalil (2015), in turn, showed a decrease in carbohydrate content in the guts of *Pheretima hawayana* earthworms exposed to TiO_2_-NPs (50 and 100 μg kg^−1^) for 28 days. In the present study, a decrease in carbohydrate levels was observed in only the highest NP treatment (1000 mg Zn kg^−1^), and only in this treatment did the earthworms exhibit weight loss during the contamination phase. In a metabolomic study on *E. fetida* exposed to different NPs, Aslund et al. (2012) proposed glucose as a bioindicator of NP exposure, while Lankadurai et al. (2015) proposed glucose and maltose as bioindicators of NP exposure (TiO_2_-NPs and C_60_-NPs, respectively). Because the total carbohydrate content was measured in the present study, we could not identify whether the decrease was due to changes in mono- or disaccharide content.

Regardless of treatment, increasing protein content was observed until the 7th day of exposure, followed by a slight decrease at days 14 and 21. A similar pattern consisting of an initial increase after a few days of exposure (between the 2nd and 4th day of exposure) followed by depletion (between the 4th and 8th day of exposure) was observed for protein levels in *Enchytraeus crypticus* exposed to 50 and 70 mg Ag kg^−1^ (EC_10_ and EC_50_ for reproduction) in ionic form in Lufa 2.2 soil (Gomes et al. 2015b). Similarly, an increase in protein content from the 3rd to 7th day was observed for *E. crypticus* exposed to EC_20_ and EC_50_ for reproduction in field soil contaminated with various forms of Cu (ions, NPs, nanowires) (Gomes et al. 2015a). Proteins are constitutive components; therefore, proteins can fuel metabolism only during extreme energy deficiency. Under moderate stress and suboptimal circumstances, metabolic compensation manifests as elevated protein turnover, which is associated with the expression of stress response proteins (Sokolova et al. 2012). The main protein groups involved in metal detoxification processes in earthworms are metallothioneins (MTs) and heat shock proteins (HSP) (Sturzenbaum et al. 1998). Elevated levels of MTs were observed in *E. fetida* after exposure to 1500 mg Zn kg^−1^ soil (Demuynck et al. 2007). Similarly, Homa et al. (2005) observed significantly increased levels of one MT isoform (w-MT2) and two HSP forms (HSP70, HSP72) in *E. fetida* coelomocytes after dermal exposure to 1.32 μg Zn cm^2^. In our study, the increase in protein levels might have also been due to elevated MT and/or HSP expression, as these proteins play a crucial role in the homeostasis, metabolism, and detoxification of metals in invertebrates (Dallinger 1996; Sørensen et al. 2003), but studying the effects of different forms of Zn on this endpoint was not the aim of this study. Surprisingly, in the present study, the lack of effect of the treatments on protein content indicates that a similar pattern of changes in protein levels over time was observed for not only all the Zn treatments but also the control. This phenomenon may be the result of the response of the earthworms to changes in the soil type. Sandy or clayey soils are not favorable conditions for earthworms (Guild 1948), and not only metal toxicity but also soil texture can affect protein content in earthworms (Beaumelle et al. 2014). It is probable that the transfer of earthworms cultured in soil rich in organic matter and with low mineral fraction into sandy loamy soil might induce skin (Spurgeon and Hopkin 1996) or gut irritation, triggering an increase in protein production.

Surprisingly, lipids were not affected by treatment or time in any of the studied phases, which suggests that earthworms can easily maintain Zn homeostasis without serious impact on this energetic component. Lipids are highly efficient storage components and are expected to be mobilized before or together with carbohydrates under toxic stress because nonprotein substrates are the preferred energy sources (Smolders et al. 2003). In previous studies, no changes in lipids were observed in the potworm *E. crypticus* exposed to 250 mg kg^−1^ (EC_10_ for reproduction) Cu ions for 8 days in Lufa 2.2 soil (Gomes et al. 2015b). Similarly, no effect of Ni ions on lipid content was observed for the isopod *Porcellionides pruinosus* exposed to 50 and 250 mg kg^−1^ Ni ions in Lufa 2.2 soil for 28 days of exposure (contamination) and after 14 days of recovery (decontamination) (Ferreira et al. 2015).

An almost significant (*p* = 0.06) effect of treatment on the total energy reserves (Ea) was observed in the contamination phase, with Ea in ZnCl_2_ 250, ZnCl_2_ 500, and ZnO-NP 1000 treatments lower by 11%, 8%, and 6%, respectively, than that in the control. In contrast, in previous studies on metal toxicity, a clear decrease in Ea has been observed in invertebrates (Moolman et al. 2007). In general, Ea was lower on days 1 and 2 than at other exposure timepoints, with maximum levels observed on days 4 and 7. The increase in Ea was mainly caused by the increased protein levels observed on days 4 and 7. Although there were no significant differences in lipid content (*p* = 0.08; Fig. 1) during the contamination phase, elevated levels of this component were observed on day 4, which might also have contributed to the increase in Ea. The increase in available energy reserves with time was also observed for *Enchytraeus albidus* exposed for 8 days to three different pesticides (dimethoate, atrazine, and carbendazim), and this increase was mainly due to increased lipid and protein levels (Novais and Amorim 2013), as observed in our study. No differences were observed in carbohydrate, protein, and lipid levels and Ea for any of the studied treatments between days 0 (before exposure), 21 (end of the contamination phase) and 35 (end of the decontamination phase) except for the ZnO-NP 500 treatment, for which the Ea was higher at day 21 than at day 0 and day 35, indicating that none of the toxicants had significant effects on the components of the energetic budget.

Zinc is an essential metal that is efficiently regulated by earthworms (Świątek et al. 2017; Gonzalez-Alcaraz et al. 2018), and when the intracellular Zn concentration exceeds the physiological level, the metal is actively pumped out or bound to zinc-binding proteins (Vallee and Falchuk 1993). Moreover, excess Zn can be sequestered intracellularly in the chloragosome granules present in the chloragogen tissue of earthworms (Morgan and Morgan 1989). Thus, the uptake, distribution, sequestration, and excretion of excess Zn can increase the energetic cost, which, in our study, manifested as an increase in Ec that was observed in all the Zn treatments in comparison to the control. Interestingly, a higher increase in Ec was observed for the ZnCl_2_ 250 and ZnO-NP 500 treatments (average increase in comparison to the control: 63% and 50%, respectively) than for the ZnCl_2_ 500 and ZnO-NP 1000 treatments (average increase in comparison to the control: 42% and 24%, respectively). The Ec increase was also observed for *E. albidus* exposed to Zn and Cd ions at EC_50_ and EC_90_ for reproduction in Lufa 2.2 soil (Novais et al. 2013). Similarly, Gomes et al. (2015b) observed an increase in Ec levels in *E. crypticus* after exposure to ionic forms of Ag at EC_10_ and EC_50_ and Cu at EC_10_ (for reproduction) between the 2nd and 4th day of exposure in Lufa 2.2 soil. As proposed by Novais et al. (2013) and Gomes et al. (2015b), the increased metabolic activity was probably associated with the costs of metal detoxification and restoration of cell homeostasis. In the present study, no differences in Ec between the EC_50_ treatments were observed (in the contamination phase), and yet, these treatments were characterized by different carbohydrate levels, demonstrating relatively high consumption of carbohydrates under exposure to relatively high nominal concentrations of Zn. On the other hand, a significantly increased Ec for the ZnCl_2_ 500 treatment compared to the other treatments was observed in the decontamination phase, indicating a strong effect of this treatment on earthworm metabolism even after completion of exposure. These subtle dissimilarities between the EC_50_ treatments in terms of energy consumption and carbohydrate metabolism, i.e., increased energy consumption even after completion of exposure in the ZnCl_2_ 500 treatment and decreased carbohydrate contents in the ZnO-NP 1000 treatment, may indirectly indicate different actions of the studied Zn forms (ionic vs. NPs), but this aspect can be further explored only by applying additional targeted cellular or molecular endpoints.

The whole-body respiration rate is an indirect measure of an organism’s maintenance costs and it is assumed that the metabolic rate should increase with increasing intoxication until irreversible effects impair metabolism itself (Calow 1991). Inhibition of oxygen consumption has been observed for earthworms exposed to metal-contaminated soil (Khan et al. 2007; Liang et al. 2017). For example, after a 3-week exposure to soil contaminated with 500 mg Zn kg^−1^, Khan et al. (2007) observed a decreased respiration rate in *Lumbricus terrestris*. Similarly, a dose-dependent decrease in the respiration rate was observed for *E. fetida* exposed to soil contaminated with nanoscale zerovalent iron (100–1000 mg kg^−1^) after 4 weeks (Liang et al. 2017). In the present study, however, the respiration rate was not affected by any of the Zn treatments. Therefore, the significant impact of exposure time manifested as constantly decreasing oxygen consumption is associated with other factors than metal stress. The respiration rate of earthworms depends on diurnal rhythms, temperature (Chuang et al. 2004), season (Phillipson and Bolton 1976), body mass (Šustr and Pižl 2009), and density (Uvarov and Scheu 2005), but the effects of all these factors were controlled, as all the measurements of respiration rate were performed during one season (summer) in a climatic chamber with a constant temperature and photoperiod. Additionally, the initial body mass of earthworms assigned to different treatments was similar. A probable explanation for the observed decrease in respiration rate over time might be the response of earthworms to changes in density. In the culture medium, earthworms were kept in groups of ca. 100 individuals per box, while in the experiment, the worms were kept individually. Uvarov and Scheu (2005) found that the respiration rate of *L. rubellus* was higher when the earthworms were kept in pairs and lower when kept individually. Considering that significant differences were already observed with such a small shift in density (from 2 individuals to 1 individual), it is highly probable that in our study, the shift from 100 individuals in the stock culture to 1 individual in the experiment forced changes in respiration that became increasingly evident over time. According to Fanslow et al. (2001), whole-body respiration is a complex physiological process that is more susceptible to experimental artifacts than Ec (ETS activity). In the present study, the direct enzymatic process of energy consumption at the cellular level (Ec) was indeed a more sensitive indicator of Zn exposure in *E. andrei* earthworms than the whole-organism respiration rate. The whole-body respiration rate at days 21 and 35 was significantly lower than that at day 0. This difference, however, probably resulted from the abovementioned overall decrease in respiration rate over time, which probably stems from factors other than the toxic effect of Zn.

## Conclusions

In general, neither different forms of Zn (Zn ions vs. ZnO-NPs) nor exposure concentrations (EC_25_ vs. EC_50_) affected the total energy reserves (Ea) or the main components of Ea (proteins and lipids), and the only clear effect of Zn was observed on carbohydrates, which were present at much lower levels in the ZnO-NP 1000 treatment than in other treatments. The exposure of *E. andrei* to Zn (both ions and NPs) caused an increase in energy consumption at the cellular level, reflecting the relatively high energy demand of responding to toxic stress, but no effect was observed at the whole-body level. In conclusion, it can be stated that *E. andrei* earthworms can cope with different forms (ions vs. NPs) of elevated Zn concentrations in soil without any serious impact on the energy budgets of the worms.

## Electronic supplementary material

ESM 1 (DOC 1307 kb)
