# Supplementary material for: Energy reserves and respiration rate in the earthworm Eisenia andrei after exposure to zinc in nanoparticle or ionic form
Source: Environ Sci Pollut Res Int. 2019 Jun 26;26(24):24933–45. doi: 10.1007/s11356-019-05753-3 (PMC6689315; doi:10.1007/s11356-019-05753-3)
Supplement: Supplementary file 1 — (DOC 1307 kb) [file 11356_2019_5753_MOESM1_ESM.doc]

**Supplementary material for:**

**Energy reserves and respiration rate in the earthworm *Eisenia andrei* after exposure to zinc in nanoparticle or ionic form**

**Environmental Science and Pollution Research**

Zuzanna M. Świątek^a^*, Agnieszka J. Bednarska^b^

^a^Institute of Environmental Sciences, Jagiellonian University, Gronostajowa 7, 30-387 Kraków, Poland

^b^Institute of Nature Conservation, Polish Academy of Sciences, Mickiewicza 33, 31-120 Kraków, Poland

***CORRESPONDING AUTHOR**

E-mail: zuza.swiatek@uj.edu.pl

**
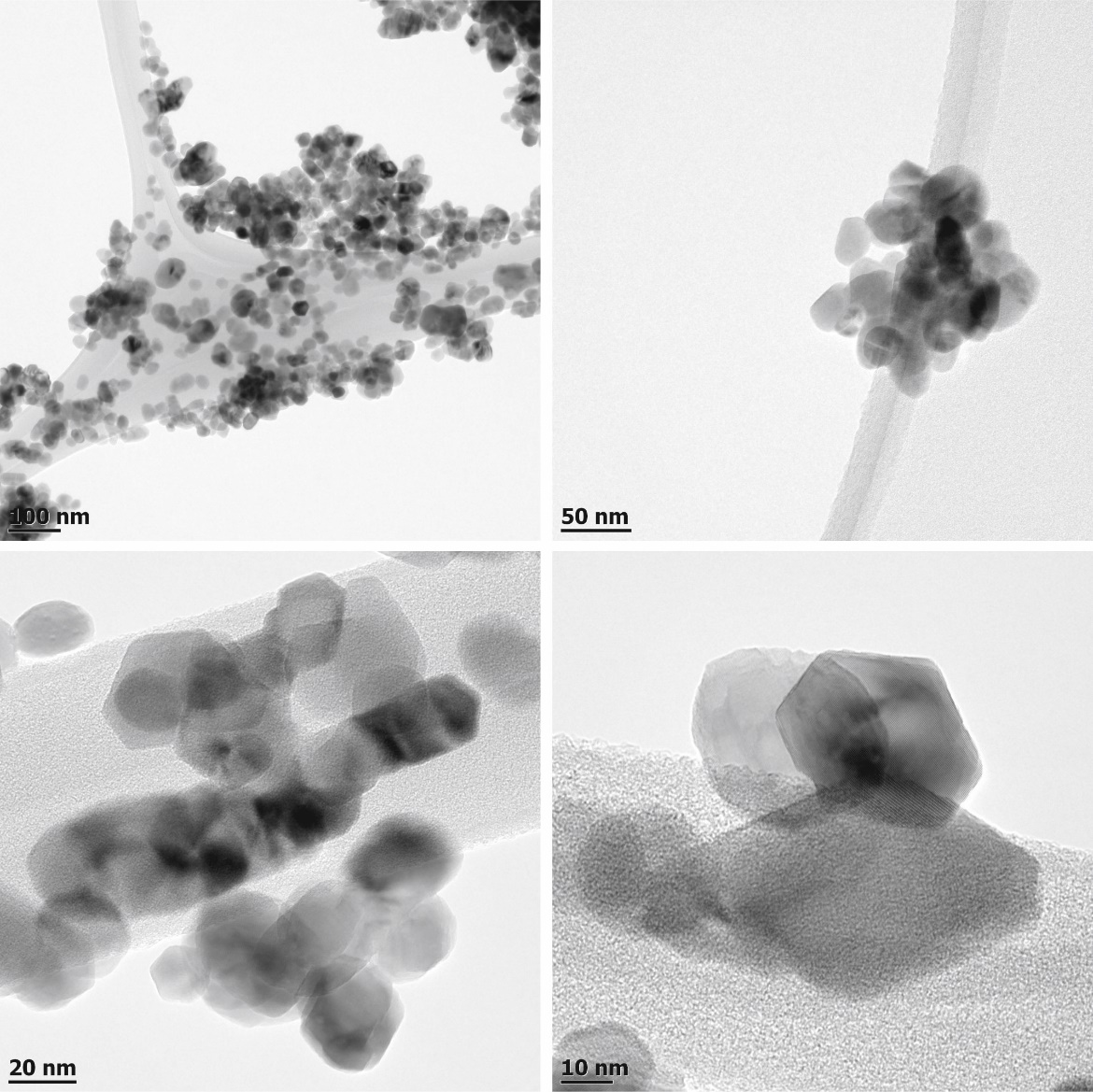
**

**Fig. S1** Transmission electron microscopy of PlasmaChem GmbH ZnO nanoparticles (ZnO-NPs), which were used to study energy reserves and respiration rate in earthworms after exposure to zinc nanoparticles or ions via soil


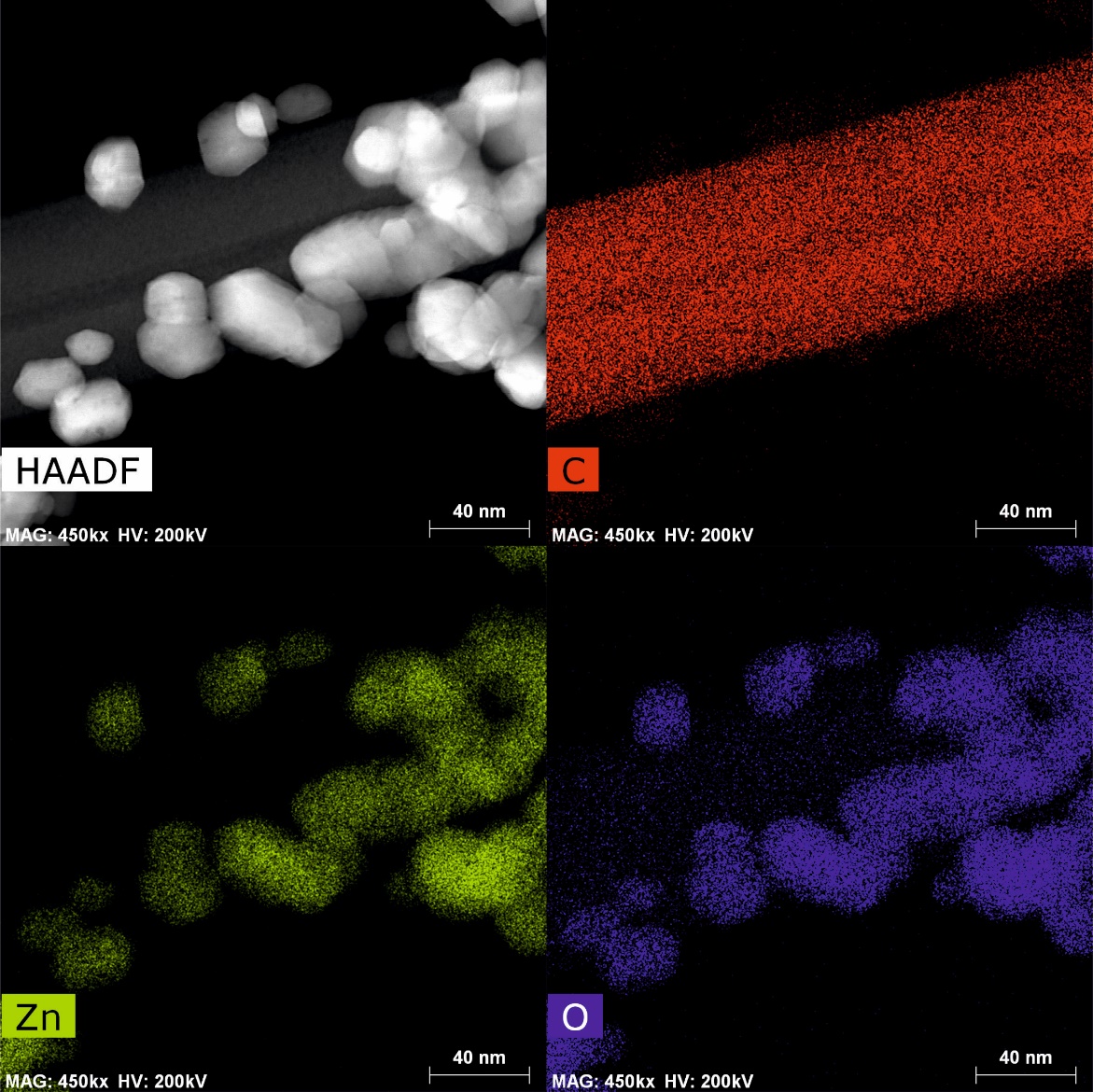


**Fig. S2** High-angle annular dark-field (HAADF) imaging of ZnO-NPs (top left), energy-dispersive X-ray mapping of background carbon film shown in red (top right), zinc from NPs shown in green (bottom left) and oxygen from NPs shown in blue (bottom right)


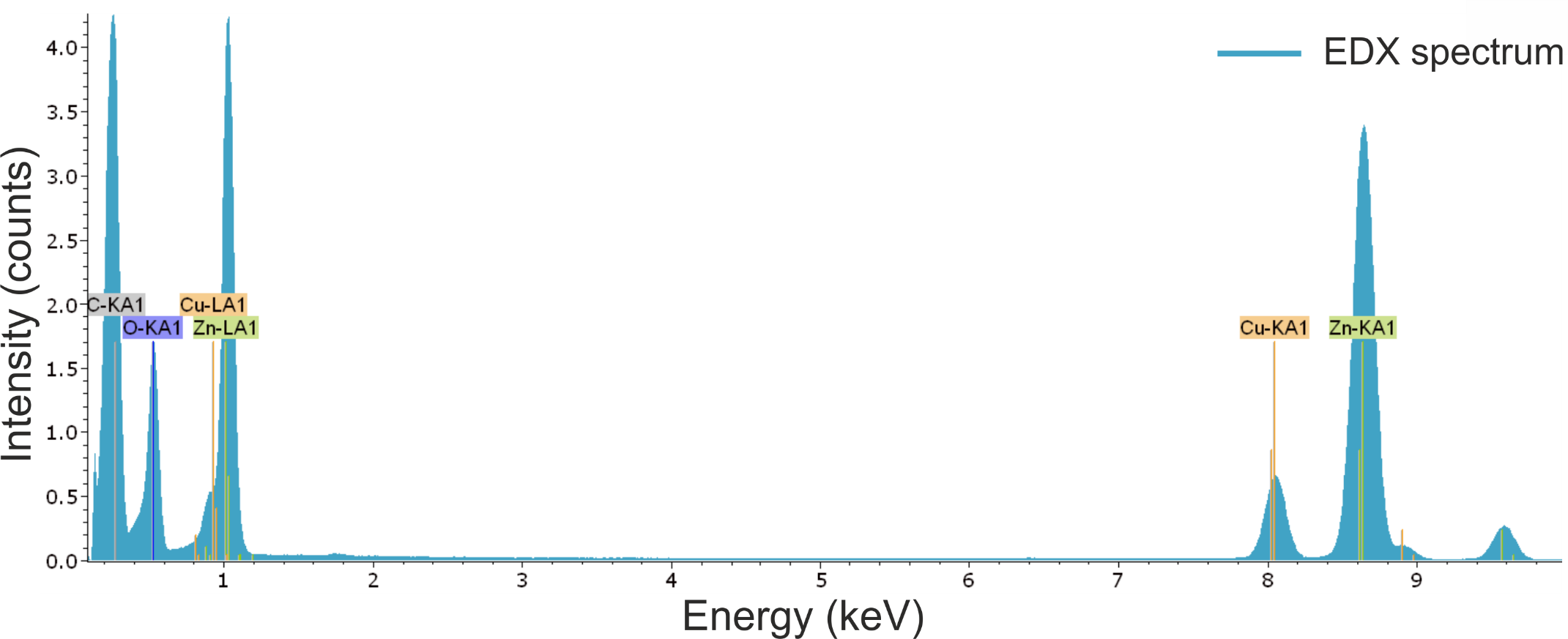


**Fig. S3** Energy-dispersive X-ray analysis of the ZnO-NPs (area from Fig. S2), showing that the NPs were composed of zinc and oxygen. Copper and carbon peaks correspond to the grid onto which the NPs were deposited


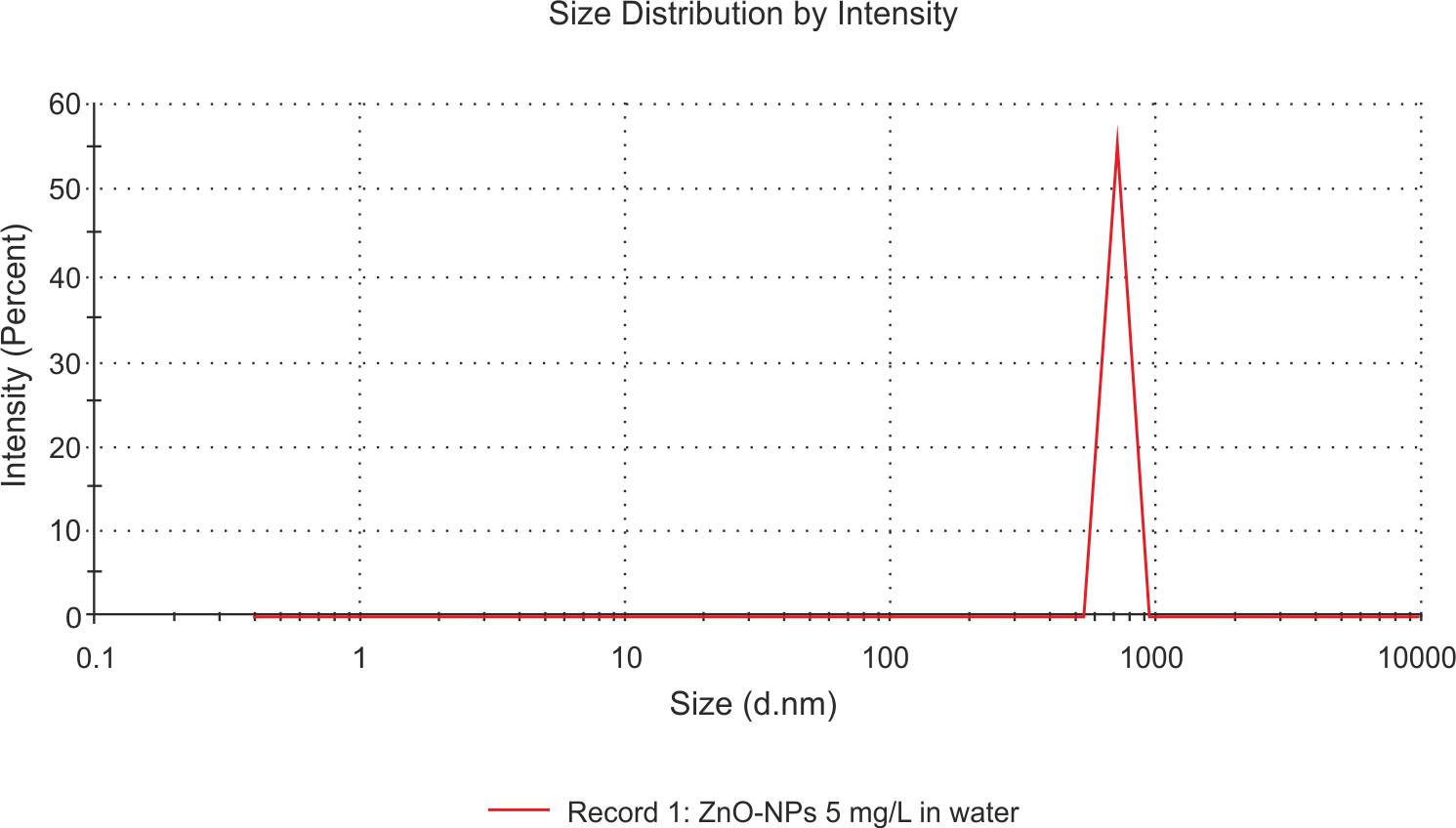


**Fig. S4** Intensity-based size distributions by dynamic light scattering analysis of 5 mg L^-1^ ZnO-NPs in deionized water


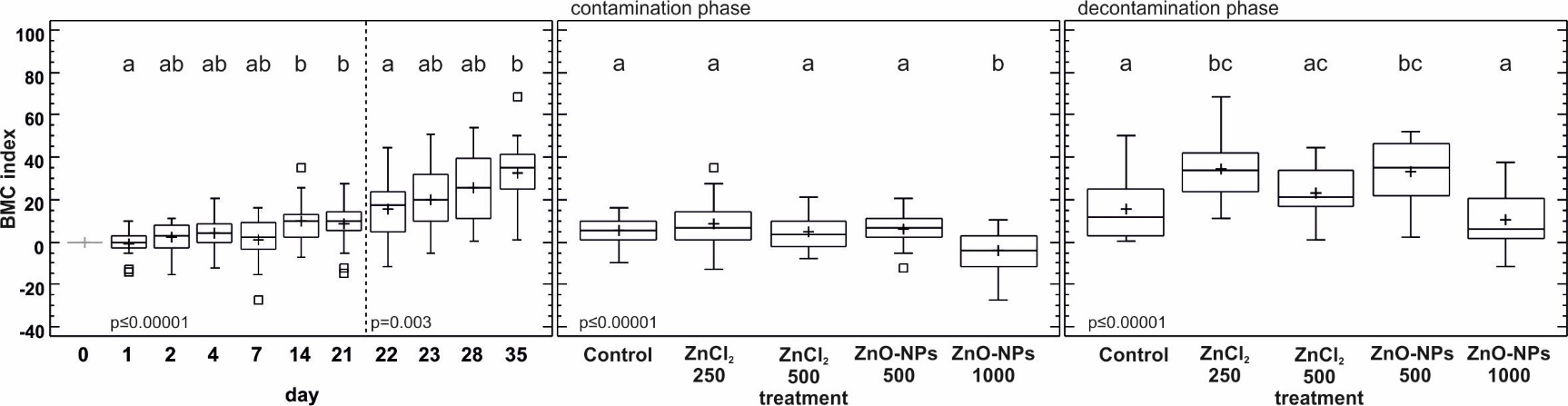


**Fig. S5** Body mass change (BMC) index of the *Eisenia andrei* earthworms exposed to Lufa 2.2 soil contaminated with different concentrations of ZnO nanoparticles (NPs) or ions (ZnCl_2_). Boxes – lower and upper quartiles, whiskers – extend to the minimum and maximum values, plus sign – mean value, center line – median, empty squares (outliers) – between >1.5 and 3 times the interquartile range. The vertical broken line indicates the start of the decontamination phase. a, b – different lowercase letters indicate significant differences between days; Kruskal-Wallis (p ≤ 0.05) test with Bonferroni 95.0% confidence level

**TABLES**

**Table S1.** ZnO-NPs characterization

| **Particle** | **Description** | **Average size (nm)** | | **Size distribution using DLS^b^ (nm)** | **Zeta-potential using DLS^b^ (mV)** |
| --- | --- | --- | --- | --- | --- |
| ZnO | Uncoated | According to manufacturer | Determined using TEM^a^ | 862.6 ± 110 | -11.1 ± 1.6 |
|  |  | 25 | 23.5 ± 7.3 |  |  |

^a^TEM – Transmission electron microscopy, N = 500

^b^DLS – Dynamic light scattering, N = 5

**Table S2.** Average (± standard deviation) zinc concentrations measured in Lufa 2.2 soil and water extracts in control soil and soil spiked with different concentrations of ZnCl_2_ or ZnO-NPs; water extracts collected at day (T): 0, 7, 14 and 21

| Treatment | Zn concentration in soil (μg g^-1^) | | Zn concentration in water extracts (μg mL^-1^) | | | |
| --- | --- | --- | --- | --- | --- | --- |
|  | Nominal | Actual | T = 0  (N = 3) | T = 7  (N = 4) | T = 14  (N = 4) | T = 21  (N = 4) |
| **Control** | **0** | 27.2 ± 1.6 | 0,06 ± 0,01 ^a^ | 0,07 ± 0,005 ^a^ | 0,05 ± 0,003 ^a^ | 0,06 ± 0,01 ^a^ |
| **ZnCl_2_** | **250** | 282.9 ± 13.6 | 0,78 ± 0,17 ^a^ | 0,61 ± 0,08 ^a^ | 0,55 ± 0,03 ^a^ | 0,60 ± 0,01 ^a^ |
|  | **500** | 538.6 ± 54.4 | 2,50 ± 0,54 ^a^ | 1,81 ± 0,15 ^a^ | 1,53 ± 0,04 ^ab^ | 1,38 ± 0,06 ^b^ |
| **ZnO-NPs** | **500** | 517.7 ± 22.0 | 1,10 ± 0,05 ^a^ | 1,14 ± 0,04 ^ab^ | 1,24 ± 0,13 ^ab^ | 1,26 ± 0,02 ^b^ |
|  | **1000** | 1062.1 ± 29.5 | 2,02 ± 0,19 ^ab^ | 1,97 ± 0,14 ^a^ | 2,06 ± 0,15 ^ab^ | 2,38 ± 0,14 ^b^ |

a, b – Different lowercase letters indicate significant differences between days within each treatment; Kruskal-Wallis (p ≤ 0.05) test with Bonferroni 95.0% confidence level.

**Table S3.** Average (± standard deviation) zinc concentrations measured in ultrafiltrates, i.e., water extracts after ultrafiltration, in soil spiked with different concentrations of ZnCl_2_ or ZnO-NPs; ultrafiltrates collected at day (T): 0, 7, 14 and 21; % - percentage of total Zn in water extracts recovered after ultrafiltration

| Treatment | Nominal Zn concentration in soil (μg g^-1^) | Zn concentration in ultrafiltrates (μg mL^-1^) | | | | % | | | |
| --- | --- | --- | --- | --- | --- | --- | --- | --- | --- |
|  |  | T = 0  (N = 3) | T = 7  (N = 4) | T = 14  (N = 4) | T = 21  (N = 4) | T = 0  (N = 3) | T = 7  (N = 4) | T = 14  (N = 4) | T = 21  (N = 4) |
| **Control** | **0** | nd | nd | nd | nd | nd | nd | nd | nd |
| **ZnCl_2_** | **250** | 0.68 ± 0.02 ^a^ | 0.40 ± 0.03 ^ab^ | 0.34 ± 0.07 ^ab^ | 0.34 ± 0.04 ^b^ | 88 | 66 | 62 | 56 |
|  | **500** | 2.37 ± 0.09 ^a^ | 1.68 ± 0.10 ^ab^ | 1.36 ± 0.04 ^ab^ | 1.25 ± 0.09 ^b^ | 95 | 93 | 84 | 88 |
| **ZnO-NPs** | **500** | 0.35 ± 0.06 ^ab^ | 0.34 ± 0.02 ^a^ | 0.40 ± 0.13 ^ab^ | 0.85 ± 0.07 ^b^ | 32 | 30 | 32 | 68 |
|  | **1000** | 0.63 ± 0.04 ^ab^ | 0.53 ± 0.07 ^a^ | 0.63 ± 0.08 ^ab^ | 1.29 ± 0.17 ^b^ | 31 | 27 | 30 | 54 |

a, b – Different lowercase letters indicate significant differences between days within each treatment; Kruskal-Wallis (p ≤ 0.05) test with Bonferroni 95.0% confidence level.

nd – not detected, Zn concentrations in ultrafiltrates of control soil were below the detection limit.

**Table S4.** Average pH_CaCl2_ (± standard deviation) of Lufa 2.2 soil spiked with different concentrations of ZnCl_2_ or ZnO nanoparticles (ZnO-NPs) and control soil, measured at days (T) 0, 7, 14 and 21

| Treatment | Nominal Zn concentration (μg g^-1^ dw) | pH in soil | | | |
| --- | --- | --- | --- | --- | --- |
|  |  | T = 0  (N = 3) | T = 7  (N = 4) | T = 14  (N = 4) | T = 21  (N = 4) |
| **Control** | **0** | 6.37 ± 0.02 ^ab^ | 6.44 ± 0.02 ^a^ | 6.07 ± 0.05 ^ab^ | 5.82 ± 0.03 ^b^ |
| **ZnCl_2_** | **250** | 6.25 ± 0.04 ^a^ | 6.33 ± 0.02 ^a^ | 6.35 ± 0.03 ^a^ | 6.36 ± 0.07 ^a^ |
|  | **500** | 5.94 ± 0.14 ^ab^ | 6.11 ± 0.02^ab^ | 5.88 ± 0.07 ^a^ | 6.17 ± 0.02 ^b^ |
| **ZnO-NPs** | **500** | 6.63 ± 0.03 ^ab^ | 6.69 ± 0.03 ^a^ | 6.63 ± 0.03 ^ab^ | 6.24 ± 0.11 ^b^ |
|  | **1000** | 6.79 ± 0.01 ^ab^ | 6.89 ± 0.02 ^a^ | 6.86 ± 0.02 ^ab^ | 6.62 ± 0.06 ^b^ |

a, b – Different lowercase letters indicate significant differences between days within each treatment; Kruskal-Wallis (p ≤ 0.05) test with Bonferroni 95.0% confidence level.
